# Unicentric Castleman Disease Involving the Submandibular Gland: A Case Report

**DOI:** 10.1155/crid/2704215

**Published:** 2026-08-13

**Authors:** Xinlong Sun, Pengfei Dong, Beining Zhang, Linkai Ma, Legang Sun, Yuliang Wang

**Affiliations:** ^1^ Department of Oral and Maxillofacial Surgery, Yantai Affiliated Hospital of Binzhou Medical University, Yantai, China, byytfy.com; ^2^ Department of Oral and Maxillofacial Surgery, Weihai Municipal Hospital, Cheeloo College of Medicine, Shandong University, Weihai, China, sdu.edu.cn; ^3^ Department of Oral and Maxillofacial Surgery, Binzhou Medical University Hospital, Binzhou, China, bzmc.edu.cn

**Keywords:** case report, diagnosis, submandibular gland, surgical excision, unicentric Castleman disease

## Abstract

This report describes a rare case of unicentric Castleman disease (UCD) involving the submandibular gland and lymph nodes. A 56‐year‐old man presented with an asymptomatic, slowly growing left submandibular mass. Imaging revealed well‐defined heterogeneous structures with prominent vascularity. Excisional biopsy of the gland and adjacent lymph nodes was performed. Histopathological examination showed features characteristic of the hyaline‐vascular variant of Castleman disease, including atrophic germinal centers with concentric “onion‐skin” layering and prominent vascularity, confirmed by immunohistochemistry. No systemic involvement was found. These findings confirmed the unicentric subtype. After complete surgical excision and 3 years of follow‐up, the patient remained disease‐free. This report underscores that UCD can occur in the submandibular region, should be included in the differential diagnosis of submandibular masses, diagnosed by characteristic imaging (well‐defined heterogeneous structures with prominent vascularity) and histopathology (hyaline‐vascular variant), and cured by complete surgical resection.

## 1. Introduction

Salivary gland tumors or lesions encompass a remarkable diversity of distinct histologies, of which tumors of glandular epithelium origin are the most common. The World Health Organization Classification of Salivary Gland Tumours latest updated in 2022 included 39 distinct entities, divided into four categories: malignant and benign epithelial tumors, nonneoplastic epithelial lesions, and mesenchymal tumors specific to the salivary glands [1, 2]. The classification newly excludes lesions or tumors that do not solely or primarily occur in the salivary glands, including hematolymphoid tumors, lipomas, hemangiomas, and nodular fasciitis [1, 3]. The most common benign nonneoplastic lesions of the submandibular glands are sialolithiasis, sialadenitis, and lymphadenitis, whereas other entities are uncommon [4]. Unicentric Castleman disease (UCD) is a rare lymphoproliferative disorder and a subtype of Castleman disease, characterized by involvement of one or more lymph nodes in one lymph node station (typically in the mediastinum, neck, abdomen, or retroperitoneum) [5]. It rarely affects the major salivary glands. To date, fewer than 10 cases of UCD involving the major salivary glands have been reported, with two case reports in the parotid gland [6, 7], three case reports in the submandibular region [8–10], and a meta‐analysis of reported cases in Taiwan and literature review that described five cases [11]. Here, we report a case of UCD occurring in the submandibular region. The case that we describe highlights three key aspects. First, it presents a case of UCD involving the submandibular gland and the regional lymph nodes and confirms that well‐defined heterogeneous structures with associated vascularity are characteristic imaging features of UCD. Second, it reaffirms that histologic examination remains the gold standard for diagnosis and that tissue sampling of suspected lesions should be performed, ideally by complete excisional biopsy. Third, it demonstrates that complete surgical excision via submandibular triangle dissection is curative for UCD in the submandibular region, as the patient remained disease‐free during the 3‐year follow‐up.

## 2. Case Report

**Figure 1:**
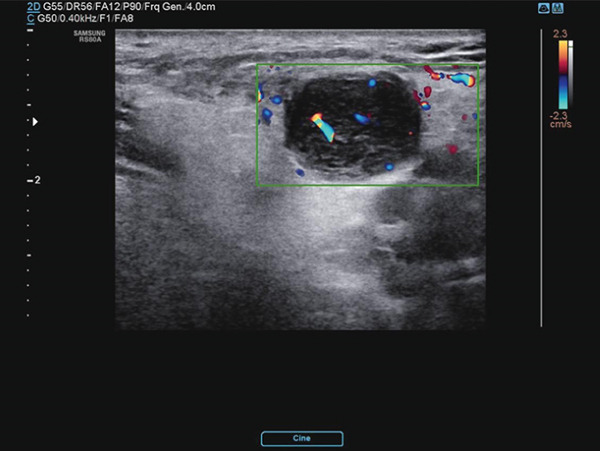
Ultrasonic manifestation showing the heterogeneous lesion with visible blood flow signals.

**Figure 2:**
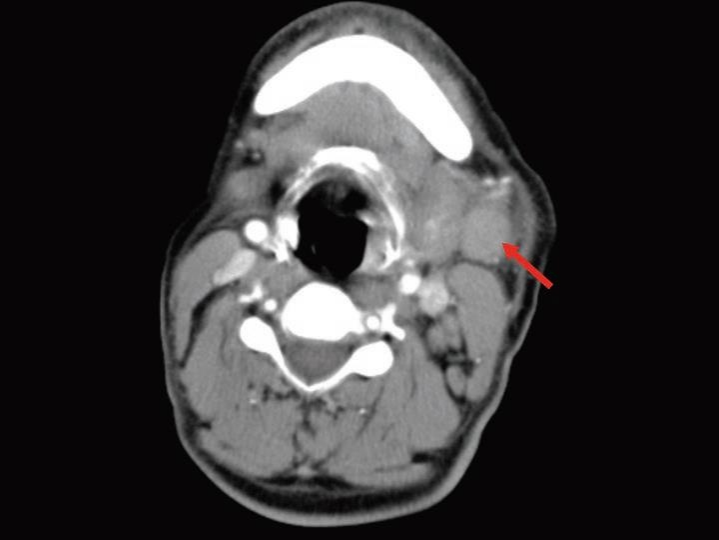
Axial slice of CT‐enhanced scanning of the lesion.

A 56‐year‐old man presented with a slow‐growing mass in the left submandibular region for about 2 years without any subjective symptoms or signs, such as pain, numbness, intermittent swelling at mealtimes, or abnormal movement of the tongue. He had no history of fever, weight loss, or dyspnea recently. He denied a medical history of hepatitis B, tuberculosis, systemic lupus erythematosus, or other infectious, autoimmune, and neoplastic diseases. He received no medical treatment during this period. Physical examination revealed that the submandibular gland was firm with multiple enlarged lymph nodes. The lesions had no tenderness or adhesion to surrounding tissues. The patient had no signs of facial paralysis, numbness, tongue deviation on protrusion, or ulceration in the oral mucosa. Ultrasound images demonstrated a heterogeneous lesion with a regular morphology, clear boundaries, approximately 2.0 × 1.3 × 1.4 cm in size, and visible blood flow signals in the submandibular glands (Figure 1). Enhanced computed tomography (CT) scanning showed the enlarged submandibular gland, multiple swollen lymph nodes with moderate and homogeneous enhancement, and associated vascularity (Figure 2). These imaging features, well‐defined heterogeneous structures with prominent vascularity, are characteristic of UCD [12]. Routine blood tests were in the normal ranges, and serological evaluations for human immunodeficiency virus (HIV), cytomegalovirus (CMV), and Epstein–Barr virus (EBV) were negative. Our initial diagnosis was a benign glandular tumor of the submandibular gland, and surgical treatment was performed. Subsequently, the dissection of the submandibular triangle, including the resection of the submandibular gland and the surrounding lymph nodes, was performed under general anesthesia.

**Figure 3:**
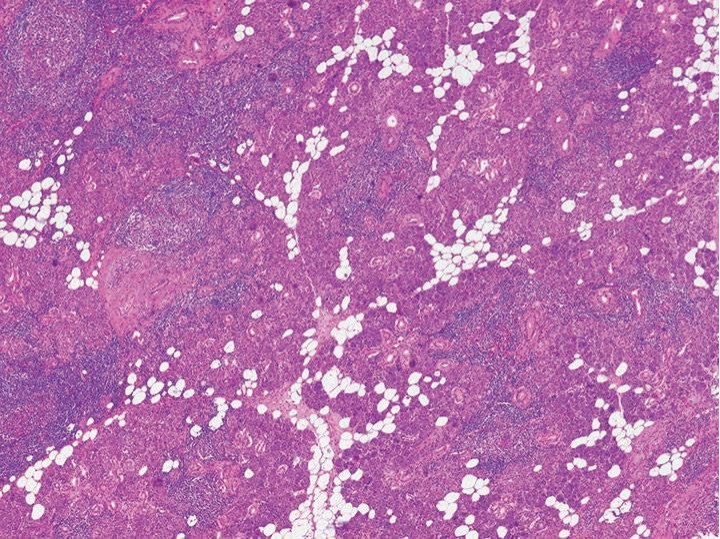
HE‐stained images of the submandibular gland (×40).

**Figure 4:**
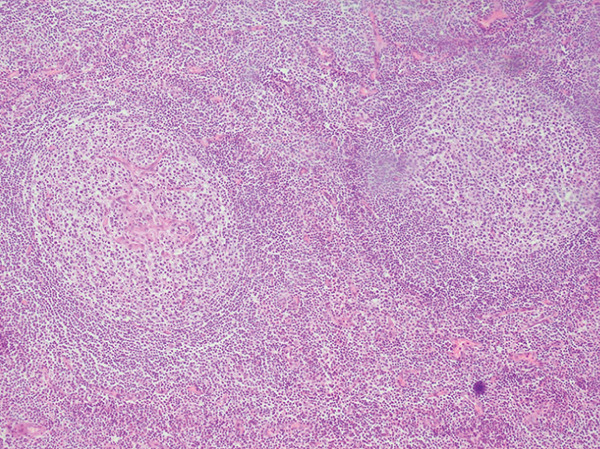
HE‐stained images of the lymph node (×100).

**Figure 5:**
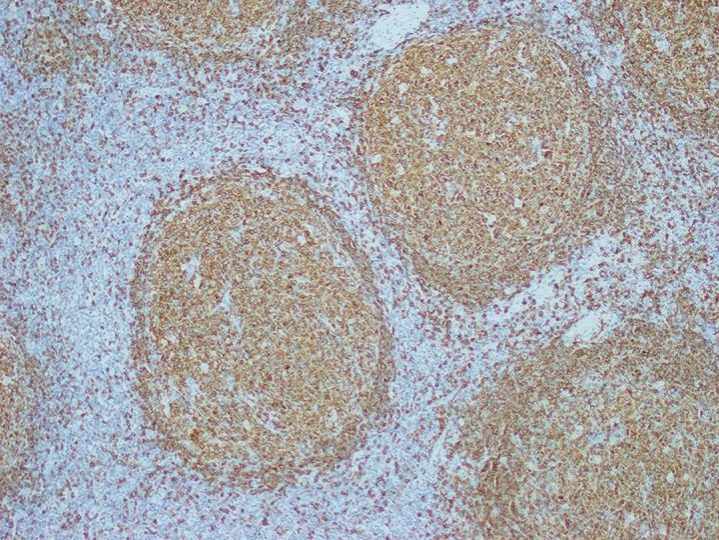
Immunohistochemistry images showing the positive staining of CD20 (×100).

**Figure 6:**
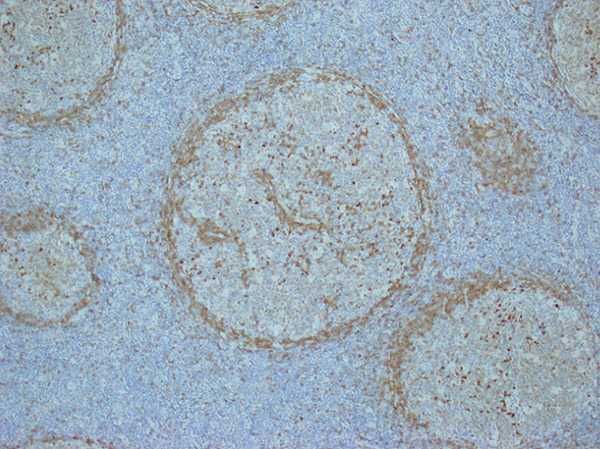
Immunohistochemistry images showing the positive staining of CD79a (×100).

The routine histopathological findings revealed the chronic inflammatory changes of the submandibular glands and multiple lymph nodes, and within nodes atrophic germinal centers, numerous germinal centers in a mantle zone, an “onion‐skin” appearance at the follicular periphery, and enhanced vascularity with hyalinization (Figures 3 and 4). On immunohistochemistry, the tumor cells were positive for CD3, CD20, CD79a, BCL‐6, CD5, CD10, and CD23, and negative for BCL‐2 and Cyclin D1 (Figures 5 and 6). The immunohistochemical panel was selected to confirm the diagnosis of Castleman disease and to exclude key differential diagnoses [13]. Positivity for CD20 and CD79a confirmed the B‐cell lineage of germinal center cells, whereas CD3 and CD5 highlighted the T‐cell population in the interfollicular areas, with no aberrant coexpression to suggest a T‐cell lymphoma [14]. CD10 and BCL‐6 positivity was restricted to germinal centers, consistent with reactive follicles. CD23 positivity highlighted disrupted and expanded follicular dendritic cell networks, a characteristic feature of the hyaline‐vascular variant [15]; lack of BCL‐2 expression ruled out follicular lymphoma (which typically shows strong BCL‐2 in germinal centers), and negative staining for Cyclin D1 excluded mantle cell lymphoma [13].

Taken together, these immunophenotypic features supported a benign, reactive lymphoproliferative process rather than a malignant lymphoma and confirmed the hyaline‐vascular subtype of Castleman disease. To definitively exclude multicentric Castleman disease (MCD) and systemic involvement, the following investigations were performed: contrast‐enhanced CT of the abdomen, pelvis, and chest, which revealed no retroperitoneal, inguinal, mediastinal, or hilar lymphadenopathy. Whole‐body positron emission tomography‐computed tomography (PET‐CT) was not performed as no suspicious findings were detected on conventional CT, and the patient had no systemic symptoms. Laboratory work‐up included complete blood count, erythrocyte sedimentation rate, serum C‐reactive protein, lactate dehydrogenase, and serum protein electrophoresis; all were within normal limits. Serological tests for HIV, EBV, and CMV were negative. These findings confirmed the unicentric subtype and ruled out MCD. The diagnosis of UCD was established. He has been followed regularly for more than 3 years with no signs of recurrence, and the data from physical examination, CT scan imaging, and laboratory studies are documented. The patient was satisfied with the treatment.

## 3. Discussion

UCD is usually characterized by the localized proliferation of one or more lymph nodes in a specific lymph node region. UCD typically manifests as one or more solitary soft‐tissue masses with well‐defined margins. Patients with UCD may be asymptomatic, exhibit symptoms associated with compression, or occasionally be accompanied by systemic symptoms (such as fever, night sweats, weight loss, anemia, etc.) or paraneoplastic pemphigus, bronchiolitis obliterans, serum amyloid type A amyloidosis [5]. Many imaging features indicate UCD, of which well‐defined heterogeneous structures with prominent vascularity are characteristic [12]. Although imaging is critical for confirming the diagnosis of UCD and ruling out the potential mimics, the histologic examination is the gold standard for UCD diagnosis. Characteristic histopathological features of UCD include a spectrum from hyaline‐vascular, which involves atrophic germinal centers and hypervascularization, to plasmacytic, which involves hyperplastic germinal centers and polytypic plasmacytosis, with a mixed group with features of both. The hyaline vascular variant is predominant in UCDs [12, 16]. Tissue sampling via full excisional biopsy is preferred; if not possible, core needle biopsy is an alternative [12, 16]. Fine‐needle aspiration is not reliable for UCD [17]. Preoperative differential diagnosis of a submandibular mass should include reactive lymphadenopathy, primary salivary gland neoplasms (such as pleomorphic adenoma or mucoepidermoid carcinoma), and lymphoma. In the present case, reactive lymphadenopathy was considered less likely given the absence of any identifiable infectious or inflammatory trigger (negative serologies for EBV, CMV, and HIV), the persistent enlargement of multiple lymph nodes over 2 years, and their homogeneous contrast enhancement on CT. Primary salivary gland malignancy was also considered, but the 2‐year history of slow growth, as well as the clinical and imaging features, were not consistent with a malignant neoplasm, which prompted further investigation.

Ultimately, histopathological and immunohistochemical findings definitively excluded these entities: the characteristic “onion‐skin” appearance and CD23+ expanded follicular dendritic cell networks confirmed UCD, lack of BCL‐2 expression ruled out follicular lymphoma, negative Cyclin D1 excluded mantle cell lymphoma, and the absence of aberrant T‐cell markers argued against T‐cell lymphoma. Thus, a systematic diagnostic work‐up integrating imaging, serology, and tissue biopsy is essential to distinguish UCD from its mimics.

UCD often affects multiple lymph nodes confined to one lymph node station. Consequently, complete dissection of the involved regional lymph nodes is required for definitive treatment. Complete surgical excision is curative for UCD, with excellent long‐term survival and low recurrence rates [5]. The submandibular triangle dissection is preferred for the treatment of UCD in the submandibular region [8–10].

## 4. Conclusion

This case demonstrates that UCD can occur in the submandibular region with involvement of the submandibular gland, an extremely rare location. Clinicians and pathologists should consider UCD in the differential diagnosis of submandibular masses. Histopathological examination remains the diagnostic gold standard, and complete surgical excision of the submandibular gland and involved lymph nodes offers an excellent long‐term prognosis. Therefore, it is essential to recognize the possibility of UCD in the head and neck region and to implement appropriate diagnostic and surgical management strategies. Multicenter case studies with long‐term follow‐up remain essential.

## Author Contributions

Xinlong Sun (co‐first author): data curation (lead) and writing—original draft (lead). Pengfei Dong (co‐first author): investigation (lead) and visualization (lead). Beining Zhang: validation (lead) and writing—review and editing (supporting). Linkai Ma: methodology (lead) and conceptualization (supporting). Legang Sun (co‐corresponding author): resources (lead). Yuliang Wang (co‐corresponding author): project administration (lead) and writing—review and editing (lead). Xinlong Sun, Pengfei Dong, Legang Sun, and Yuliang Wang have contributed to the work equally and should be regarded as co‐first authors.

## Funding

No funding was received for this manuscript.

## Disclosure

All authors have read and approved the final version of the manuscript. The corresponding authors had full access to all of the data in this study and takes complete responsibility for the integrity of the data and the accuracy of the data analysis.

## Ethics Statement

Ethical approval was not required for this single anonymized case report. According to the regulations of our institution, ethics approval is not required for retrospective case reports involving a single patient when all patient identifiers have been removed, and the study does not alter or interfere with routine clinical practice. Written informed consent was obtained from the patient for publication of this case report and any accompanying images. A copy of the signed consent form is retained by the corresponding authors and is available upon request. This report was prepared in accordance with the Declaration of Helsinki.

## Conflicts of Interest

The authors declare no conflicts of interest.

## Supporting information


**Supporting Information** Additional supporting information can be found online in the Supporting Information section. 

## Data Availability

The data that support the findings of this study are available on request from the corresponding authors. The data are not publicly available due to privacy or ethical restrictions.
