# Supplementary material for: Unicentric Castleman Disease Involving the Submandibular Gland: A Case Report
Source: Case Rep Dent. 2026 Aug 13;2026:2704215. doi: 10.1155/crid/2704215 (PMC13471636; doi:10.1155/crid/2704215)
Supplement: Supplementary file 1 [file CRID-2026-2704215-s001.pdf]

CARE case report guidelines

CARE Checklist of information to include when writing a case report

(cc) BY-NC-ND

Topic	Item	Checklist item description	Reported on Line
Title	1	The diagnosis or intervention of primary focus followed by the words "case report"	1
Key Words	2	2 to 5 key words that identify diagnoses or interventions in this case report, including "case report"	36-37
Abstract (no references)	3a	Introduction: What is unique about this case and what does it add to the scientific literature?	56-64
	3b	Main symptoms and/or important clinical findings	72-84
	3c	The main diagnoses, therapeutic interventions, and outcomes	85-88 ,117-119
	3d	Conclusion—What is the main "take-away" lesson(s) from this case?	160-168
Introduction	4	One or two paragraphs summarizing why this case is unique (**may include references**)	56-64
Patient Information	5a	De-identified patient specific information.	66-67
	5b	Primary concerns and symptoms of the patient.	66-69
	5c	Medical, family, and psycho-social history including relevant genetic information	68-71
	5d	Relevant past interventions with outcomes	71-72
Clinical Findings	6	Describe significant physical examination (PE) and important clinical findings.	72-75
Timeline	7	Historical and current information from this episode of care organized as a timeline	66-120
Diagnostic Assessment	8a	Diagnostic testing (such as PE, laboratory testing, imaging, surveys).	75-84
	8b	Diagnostic challenges (such as access to testing, financial, or cultural)	84-85 , 89-104
	8c	Diagnosis (including other diagnoses considered)	84-85 , 89-104
	8d	Prognosis (such as staging in oncology) where applicable	117-119
Therapeutic Intervention	9a	Types of therapeutic intervention (such as pharmacologic, surgical, preventive, self-care)	85-88
	9b	Administration of therapeutic intervention (such as dosage, strength, duration)	85-88
	9c	Changes in therapeutic intervention (with rationale)	66-119
Follow-up and Outcomes	10a	Clinician and patient-assessed outcomes (if available)	117-119
	10b	Important follow-up diagnostic and other test results	117-119
	10c	Intervention adherence and tolerability (How was this assessed?)	117-119
	10d	Adverse and unanticipated events	117-119
Discussion	11a	A scientific discussion of the strengths AND limitations associated with this case report	167-169
	11b	Discussion of the relevant medical literature **with references**.	51-64 , 96-99
	11c	The scientific rationale for any conclusions (including assessment of possible causes)	160-165
	11d	The primary "take-away" lessons of this case report (without references) in a one paragraph conclusion	165-167
Patient Perspective	12	The patient should share their perspective in one to two paragraphs on the treatment(s) they received	119-120
Informed Consent	13	Did the patient give informed consent? Please provide if requested	Yes ☑ No ☐
